# Fatty Acids Quality in Middle Eastern Traditional Dishes, Arabic Sweets and Market Foods Frequently Consumed in Lebanon

**DOI:** 10.3390/nu13072462

**Published:** 2021-07-19

**Authors:** Maha Hoteit, Edwina Zoghbi, Alissar Rady, Iman Shankiti, Ayoub Al-Jawaldeh

**Affiliations:** 1 Faculty of Public Health, Lebanese University, Beirut 6573, Lebanon; m.hoteit@ul.edu.lb; 2 Country Office for Lebanon, World Health Organization, Beirut 5391, Lebanon; zoghbie@who.int (E.Z.); Radya@who.int (A.R.); shankitii@who.int (I.S.); 3 World Health Organization Regional Office for the Eastern Mediterranean, Cairo 11371, Egypt

**Keywords:** unsaturated fatty acid, saturated fatty acid, monounsaturated fatty acid, traditional dishes, Lebanon

## Abstract

The prevalence of diet-related non-communicable diseases is on the rise in the countries of the Eastern Mediterranean, including Lebanon. This study aimed to provide data on fatty acid profiles and ratios of Lebanese composite dishes, Arabic sweets, and market foods. Methods: Thirty types of traditional dishes, collected from five different Lebanese governorates, thirty-seven types of Arabic sweets and forty-six market food products were considered for analysis. Food samples were chemically analyzed for total, unsaturated and saturated fatty acids. The range of total fatty acids in composite dishes, Arabic sweets, and market food products was between 1.2–11.7 g/100 g, 5.3–25.8 g/100 g, and 0.5–100 g/100 g, respectively. Additionally, the range of saturated fatty acids in composite dishes, Arabic sweets, and market food products was between 0.5–4.9 g/100 g, 2.5–23.6 g/100 g and 0.1–56.4 g/100 g, respectively. Furthermore, about 75% of these foods were poor in unsaturated fatty acids. Regarding saturated fatty acid, the polyunsaturated to monounsaturated (P.M.S) ratio was lower than the recommended ratio of 1:1:1 in 96% of samples. To conclude, there is a need to prioritize fat content in foods and consider processing modifications in the food production system with the aim of achieving a higher P:M:S ratio intake among the population.

## 1. Introduction

Diet Westernization and urbanization have led to an expansion in out-of-home meal consumption in low-middle income countries (LMICs) [1]. Nowadays, Eastern Mediterranean (EM) countries are facing rapid transitions in food patterns and development that have caused a shift in the prevalence of non-communicable diseases (NCDs) [2]. In 2018, half the worldwide population were residing in cities, and it is expected that two-thirds of humans will be urban by 2050 [3] According to the Lebanon demographics profile report in 2019, 89% of the total Lebanese population was urban [4]. In Lebanese citizens, diet quality [5] and food patterns [6] were affected by this shift in urbanization. According to a study published in 2003, a representative sample of 566 Lebanese adults from five areas in Lebanon, aged 20–85 years, showed that less than 60% of the population consumed more than seven traditional dishes per week [7]. It was also observed that the majority of the Lebanese population forswears traditional cooking patterns in favor of fast-food dietary patterns [8]. The triple helix of diet, lifestyle, and chronic diseases is linked to dietary patterns and this association is well established [9]. Saturated fatty acids (SFA) that contain single carbon with carbon bonds are found in many animal and plant sources such as meat, ghee, margarine and chocolate. Concerning the monounsaturated fatty acids (MUFA), the predominant type is cis oleic acid (18:1). The following sources contain MUFA in abundance: nuts, avocados, and olive oil (up to 80% MUFA). MUFAs present several health benefits, such as anti-inflammatory and cardioprotective effects and lipid profile improvement [10]. Additionally, evidence has been reported that a MUFA-rich diet instead of a high carbohydrate diet enhances the glucose tolerance of type 2 diabetes patients [11]. Regarding the polyunsaturated fatty acids (PUFA), they are essential fatty acids which are not synthesized in the body. However, PUFAs are important to be added in the diet in order to maintain good health. There are many forms of PUFAs, of which linolenic and linoleic acids are the most discussed [12,13]. The predominant PUFA is found in the form of linoleic acid (18:2; *n* − 6) which is present in vegetable oil, nuts, seeds, meats, and eggs. A lower proportion of PUFA is in the form of alpha-linolenic acid (18:3; *n* − 3); it is found in oily fish and green leafy vegetables [12]. There is a limited number of studies on fatty acid intake and ratios in the Eastern Mediterranean Region (EMR) [14]. Furthermore, to tackle non-communicable diseases, the planning of nutritional interventions is imperative, yet needs an assessment of the food dietary patterns and intakes of the population. However, very few dietary data instruments related to traditional dishes, Arabic sweets, and market foods intake are available in Lebanon. Thus, in response to the need for the development of culturally specific data on fatty acid content and ratios among Eastern Mediterranean citizens in order to assist healthcare professionals in evaluating the usual dietary intakes of fatty acids, this study was initiated.

## 2. Materials and Methods

### 2.1. Food Selection

A range of traditional dishes, Arabic sweets, and market foods were identified and selected for nutrient analysis. Thirty types of traditional dishes composed of many food groups, such as meats, fish, cereals, legumes, vegetables and fats, and thirty-seven types of Arabic sweets, composed of nuts, seeds, fats, cereals, dairy and sugar groups, were selected based on their frequency of consumption by the Lebanese population [8,15]. In addition, forty-six market foods were randomly selected from supermarkets regularly visited by the citizens in Beirut and Mount Lebanon areas. These foods ranged from dessert items, bakery products, local and imported on the shelf market products, coffee, and nuts. The definition of traditional ‘composite dishes’ is found in a previous study [16]. The names of the dishes most eaten by Lebanese citizens and chosen for this study are presented in Table 1 and their ingredients were listed previously [16]. Similarly, the lists of Arabic sweets and market foods are presented in Table 2 and Table 3.

The full methodology of food list identifications and food sampling is described elsewhere [16,17].

### 2.2. Chemical Analysis

Around 500 g of each sample was mashed, then analyzed, and the remaining samples were kept frozen at −18 °C for further analysis. The fatty acid profile was measured using gas chromatography. Each analysis method was selected considering guidance from the technical committee at the Industrial Research Institute laboratories in Beirut and following standardized protocols. The Association of Official Analytical Chemists (AOAC) methods were used for analysis of nutrients in food matrices [18].

Soxhlet extraction (Total fatty acids extraction):

The Roese–Gotlieb method was used in the investigation of the fat content (AOAC 945.48, 933.05 & 963.15, 2019). Between 1–2 g of the dried food sample was filtered using a piece of filter paper. Later, it was wrapped and introduced into the soxhlet thimble. To avoid sample spilling, a cotton plug was placed at the top of the thimble. The soxhlet apparatus was assembled and a light petroleum, hexane, heptane, diethyl ether, or cyclohexane was used. The extraction was performed overnight. If solids from the thimble or sample were found in the solvent extract, a filtration before evaporation into another tarred flask or beaker was performed. Then it was dried to a constant weight, which was attained when successive 1 h drying periods showed additional loss of less than 0.05 fat. The percentage (%) fat was equal to (g) fat × 100 g sample.

Fatty Acid Profile (saturated, unsaturated, trans):

The extracted fat of the sample that was obtained during fat determination was used to analyze the fatty acid profile. Between 200–500 mg of the lipid sample was placed in a boiling flask with chips; then, 5 mL of 0.5M methanolic KOH was added. Esterification was performed by boiling under reflux for 3–5 min. An addition of 15 mL of esterification reagent (2 g NaNH_4_ + 60 mL methanol + 3 mL conc sulfuric acid) through the condenser was performed, and then the sample was boiled for 15 min. After cooling, there was an addition of 50 mL of distilled water and 25 mL of the solvent. The organic layer was isolated by means of a separatory funnel. Finally, the solvent layer was washed twice with distilled water.

Chromatographic analysis:

Column: fatty acid methyl esters (FAME) length: 30 m, 0.32 ID
Injection volume: 1 µL
Injector temperature (PTV): injection: 60 °C for 0.1 min
Transfer: ramp 10 °C/min to 270 °C, 1 min hold
Carrier flow (He): 2.0 mL/min
Split flow: 20 mL/min (split ratio: 20)
Detector temperature (flame ionization detection (FID)) 280 °C
Detector gases flows: Air 350 mL/min, Hydrogen 32 mL/min, Make-up (N_2_) 30 mL/min

Oven Program:

Initial temperature: 100 °C, hold 3 min
-Ramp 1: 10 °C/min to 200 °C, hold 3 min
-Ramp 2: 10 °C/min to 250 °C, hold 5 min

Using the chromatograph software, an integration of the areas under the peak was detected under the standards peaks. A calculation as percentage areas was done.

The sum of SFA, MUFA, and PUFA, as well as the sum of trans fatty acids, was calculated accordingly (ISO 12966-4, AOAC 985.21, 2019).

### 2.3. Fatty Acids Ratios

The polyunsaturated:saturated (P:S) fatty acids ratio is frequently used as an indicator of the quality of fat in the diet. The United State (U.S.) National Research Council recommends a P:S ratio of 1:1. Furthermore, the U.S. Senate Select Committee recommended a ratio of 1:1:1 of PUFA, MUFA, and SFA (P:M:S) in the dietary fat intake in the United States [19]. Since these dishes constitute the major food items in lunches and dinners, both the P:S and P:M:S ratios were calculated and are presented in Table 1, Table 2 and Table 3. Additional calculations of M:S and P:M ratios are presented in the same tables, but are not discussed in this article.

### 2.4. Contributions to Daily Values

The daily value (DV) is defined by the US Food and Drug Administration (FDA) as the following: “reference values for reporting nutrients on the nutrition labels”. Moreover, the percentage (%) of DV assists the consumer in recognizing whether the serving of food and its content in nutrients is high (>20%), good (10–19%), or low (<10%) [17,20]. According to the “Healthy Diet report” published by the World Health Organization [21], in a total daily calorie intake of 2000 Kcal, the total fatty acids, MUFA, PUFA and SFA daily intake should constitute 35%, 20%, 10% and up to 9%, respectively. In term of grams per day (g/d), these percentages are equivalent to 78 g/d, 44 g/d, 22 g/d and 20 g/d, respectively. Referring to the dishes and food items selected in our study, the required amount per day of total, unsaturated, and saturated fatty acid was calculated. The calculations are displayed in Table 1, Table 2 and Table 3.

## 3. Results and Discussion

The results of the fatty acids profile, DVs and ratios of the Lebanese composite dishes, Arabic sweets and market products are presented in Table 1, Table 2 and Table 3.

### 3.1. Total Fatty Acids

The range of total fatty acids in composite dishes, Arabic sweets, and market food products was between 1.2–11.7 g/100 g, 5.3–25.8 g/100 g, and 0.5–100 g/100 g, respectively. Around only 16% of traditional dishes samples contained an amount of total fatty acids that exceeded the 10% of DV. The highest mean value of total fatty acids was observed in Shawarma Lahma and Falafel (Table 1). Other dishes had variable content of total fatty acids that depended on the fat source in each recipe. According to literature and in terms of Falafel, our results went hand-in-hand with those of Bawadi & Al-Sahawneh in Jordan [22] and Al Faris in Saudi Arabia [23] (Table 4). As for fatayer sabanikh, in our results, the total fatty acids were much higher than that reported in Lebanon in 1970 [24] in Jordan [22], in Saudi Arabia [23] and in Bahrain [25,26] (Table 4). Furthermore, hindbe bil zeit and lahm bil ajin showed that the first dish contained 1.5 times more and the second dish contained 5 times less total fatty acids compared to 1970, respectively [24]. The type of frying oil, the fatness of meat and the cooking method of the composite dishes all together could play a major role in the variability of our results.

Regarding the Arabic sweet samples, around 48% of the samples contained a high amount of total fatty acids in which their contributions to the DV exceeded 20%. The highest mean value of total fatty acids was observed in halawa (25.8 g/100 g) (Table 2). Also, a high content of total fatty acids was detected in halawa light (25.5 g/100 g), barazik (25.3 g/100 g), kallaj kashta (25.1 g/100 g), saniora (23.8 g/100 g), baklava mixed (23.4 g/100 g), baklava mixed light (20.5 g/100 g), ghourayba (20.4 g/100 g), and moushabak (20.1 g/100 g) (Table 2). Referring to the literature, and comparing with our results, the total fatty acids in baklava mixed was much lower than that reported in Jordan [22], and in Bahrain [25,26] (Table 4). Similarly, for barazik and ghourayba, their content in total fatty acids was lower that reported in Jordan [22] (Table 5). However, our results for kallaj kashta go with the results of Bawadi & Al-Sahawneh in Jordan [22] (Table 4).

As for the market foods, the total fatty acid content ranged between 0.5 g and up to 100 g in 100 g of the analyzed market foods. The lowest total fatty acid content was observed in the baguette and tuna packed in water (0.5 g/100 g) while the highest content was observed in corn oil, olive oil, and sunflower oil (100 g/100 g) (Table 3).

### 3.2. Saturated Fat

The highest mean values of SFA were present in Shawarma lahma (Table 1). As for the Arabic sweets, the highest mean values of SFA were observed in kallaj kashta (23.6 g/100 g), saniora (16.6 g/100 g), and maamoul mad joz (15.8 g/100 g) (Table 2). The SFA content varied across market foods ranging between 0.5% to more than 50% (0.1 and 56.4 g/100 g in the baguette and butter respectively). In addition, half the foods had a % DV higher than 20% except for tuna packed in oil and water and some baked goods (Table 3).

### 3.3. Monounsaturated Fatty Acid

The highest mean value of MUFA was observed in hindbe bil zeit (6.3%) (Table 1). Half the traditional dish samples met less than 5% of the DV for MUFA and as such were considered poor sources of MUFA (Table 1). Additionally, the highest mean value of MUFA among the Arabic sweets was seen in daoukia (13.6 g/100 g) (Table 2). However, the MUFA contribution was less than 14% in the majority of the Arabic sweets. Similarly, almost all market products contained trace amounts of MUFA except for olive oil (74.3 g/100 g) (Table 3).

### 3.4. Polyunsaturated Fatty Acid

Fatayer sabanikh had the highest mean value of PUFA across the Lebanese governorates (Table 1). Only three traditional dishes out of 30 met more than 20% DV for PUFA; therefore, the majority of these foods were considered poor sources of PUFA (Table 1). The highest mean value of PUFA was seen in madlouka which contains 3.4 g/100 g (Table 2). The majority of the Arabic sweets presented a poor PUFA content. Also, almost all market products contained trace amounts of PUFA except for sunflower Oil (62.6 g/100 g) (Table 3).

According to Brouwer (2016), an amelioration in the lipoprotein profile could be observed when substituting SFA in the diet with PUFA; this same effect was seen slightly with MUFA [28]. According to Zong, et al., MUFA affects serum lipid levels and induces benefits at the heart level [29]. The poorness in MUFA and PUFA of the majority of traditional dishes, Arabic sweets and market food in Lebanon is an alarming issue that need to be studied in light of the rise of NCDs, which are responsible of 91% of deaths in Lebanon [30].

### 3.5. Fatty Acids Ratios

#### 3.5.1. Polyunsaturated Fatty Acid: Saturated Fatty Acid Ratio (P:S)

Fatayer sabanikh was seen to have the most elevated mean value of P:S ratio (Table 1), whereas the P:S ratio of all the meals was far from 1:1, indicating a low-fat quality diet. This may be due to the heavy usage of animal fat sources in foods instead of using vegetable oils, and it highlights the need for national food preparation adjustments. Furthermore, almost all Arabic sweets have shown a deviation of P:S ratio from 1:1 (Table 2). Furthermore, the P:S ratio in all the market foods was also deviating from 1:1 (Table 3).

#### 3.5.2. Polyunsaturated Fatty Acid: Monounsaturated Fatty Acid: Saturated Fatty Acid Ratio (P:M:S)

The P:M:S across all food samples from the different governorates deviated from the recommended 1:1:1 ratio. Also, a deviation of the P:M:S ratio from 1:1:1 was seen among all Arabic sweets (Table 2), as well as among all market foods (Table 3).

These findings further highlight the need to modify traditional dishes in terms of fatty acids to achieve a more calibrated P:M:S ratio. This deviation could lead to an inverse relationship between the unsaturated fatty acids levels in food and coronary heart diseases [28]. A deviated P:M:S ratio can raise serum cholesterol and impact heart disease risk markers [31]. The deviated ratio in our study went hand in hand with Sawaya, et al. in Kuwait [32].

### 3.6. Comparison Between Market Foods: Nutrient Content Versus Nutrition Fact Label

All products were found to have discrepancies in reporting the actual nutrient content when compared to their respective nutrition facts label (Table 5). For instance, the butter light had a difference between the analyzed nutrition (61.5 g/100 g) and the labelled nutrition (20 g/100 g). Some products such as sunflower oil and olive oil had matching values for fatty acid content. As for the saturated fatty acid content, there was a discrepancy between the nutrition label and nutrient content of the chocolate wafers-brand 2 (19.1 g/100 g and 9 g/100 g), chocolate with milk (36.1 g/100 g and 46 g/100 g), chocolate-dark (33.1 g/100 g and 23.3 g/100 g), potato chips (20.2 g/100 g and 10 g/100 g) and butter light (43.4 g/100 g and 53.3 g/100 g). Nonetheless, there were analyzed values double or more than that of the labelled in products including Lays chips cheese and potato chips light (Table 5).

While MUFA and PUFA were rarely reported on the nutrition facts label, analysis of the market food products revealed trace amounts as mentioned earlier. In terms of promoting healthy behaviors and food choices, not only the nutritional quantity of food consumed, but also the quality of ingredients including the lipid profile, needs to be considered, because according to a recent systematic review, there is an impact of food labels on food choices and healthiness attitudes and practices among consumers [33].

There is a gap in the data on food consumption patterns among Lebanese populations and this should be identified and quantified by means of individual dietary surveys, without which the dietary intake of fatty acid cannot be estimated. Nowadays, such an approach is a challenge in LMICs, which are grasping the nettle of infectious diseases and epidemic outbreaks (such as Ebola and COVID-19), removing the focus from acute and chronic care of NCDs. Thus, long-term care for NCDs would assist in making the health systems ready to walk side by side with other acute diseases. The implementation of such a system will require fund allocations at both the national and international level. Moreover, this achievement should align the NCD agenda, taking into consideration national and international efforts to implement accessible and equitable health promotion systems in order to fortify capacity building and prioritize the achievement of NCD-focused health systems.

## 4. Conclusions

For the first time, this study shows that the majority of the frequently consumed traditional dishes in Lebanon have a deviated P:M:S ratio reflecting an alarming impact on coronary health of the Lebanese population and ringing the alarm bell regarding the priority of controlling the quality of Lebanese diet in terms of fatty acids. The results of this study recommend that, although cultural heritage is encouraged by consuming traditional dishes, a healthier choice of ingredients will go a long way in preventing non-communicable diseases associated with an increased consumption of SFA. There is additionally a requirement for creating national and international databases on the dietary fat intake in all age categories. Such information, which is as yet missing, will be very useful in considering the connection between dietary components and the etiology of NCDs in Lebanon.

## Figures and Tables

**Table 1:** Fatty acid ratios and total fatty acid mean values in 100 g of edible portions of traditional dishes and the percentage of their daily contribution in a 2000 Kcal-diet.

Dish	Mean Values				Mean Values				Mean Values			
	Amounts in 100 g of Edible Portions				Percentage of Daily Contribution in 2000 Kcal Diet				Fatty Acid Ratios			
	Fat	SFA	MUFA	PUFA	Fat	SFA	MUFA	PUFA	P:S	M:S	P:M	P:M:S
Baba ghanouj	9.4	4.3	4.2	0.7	12.1	21.9	9.5	5.8	0.1	0.9	0.1	0.1:0.9:1
Batata mahchi	1.2	0.5	0.3	0.2	1.5	2.8	0.8	1.9	0.4	0.6	0.6	0.4:0.6:1
Borgul bi banadoura	5	2.4	1.6	0.9	6.4	12.3	3.6	6.9	0.3	0.6	0.5	0.3:0.6:1
Chichbarak	4.6	3.1	1	0.3	5.9	15.9	2.3	2.5	0.1	0.3	0.3	0.1:0.3:1
Falafel	11.7	4.4	5.4	1.7	15	22.4	12.2	13.6	0.3	1.2	0.3	0.3:1.2:1
Fatayer sabanikh	11.1	2.5	4	4.5	14.3	12.7	9.2	34.7	1.7	1.6	1.1	1.7:1.6:1
Fattat Hommos	7	3.6	2.8	0.5	9	18	6.4	4.3	0.1	0.7	0.2	0.1:0.7:1
Fattoush	2.4	1.7	0.9	0.2	3.7	8.7	2.1	1.6	0.1	0.5	0.2	0.1:0.5:1
Foul moudamas	3.4	1.5	1.7	0.1	4.4	7.7	3.9	1.3	0.1	1.1	0.1	0.1:1.1:1
Hindbe bil zet	10.7	3	6.3	1.3	13.7	15.2	14.3	10.2	0.4	2	0.2	0.4:2.:1
Hommos bi tahini	6.4	2.8	2.7	0.8	8.2	14.2	6.1	6.5	0.3	0.9	0.3	0.3:0.9:1
Kafta wa batata	6.3	3.7	1.9	0.5	8.1	18.7	4.4	4.1	0.1	0.5	0.2	0.1:0.5:1
Kebba bil sayniya	6.4	3.7	1.9	0.6	8.2	18.6	4.4	5.1	0.1	0.5	0.3	0.1:0.5:1
Koussa mahchi	2.4	1.4	0.5	0.3	3.1	7.4	1.3	2.5	0.2	0.3	0.5	0.2:0.3:1
Lahm bil ajin	8.9	2.8	3.1	2.9	11.4	14.2	7.1	22.7	1	1.1	0.9	1:1.1:1
Loubia bil zet	5.6	1.4	2.7	1.4	7.2	7.3	6.1	11.3	1	1.8	0.5	1:1.8:1
Malfouf mahchi	2.1	1.1	0.6	0.3	2.7	5.6	1.4	2.5	0.3	0.5	0.5	0.3:0.5:1
Moujadara	5.8	1.4	2.5	1.6	7.4	7.4	5.8	12.7	1.1	1.7	0.6	1.1:1.7:1
Moghrabia	3.9	2.2	1.2	0.4	5	11	2.8	3.2	0.1	0.5	0.3	0.1:0.5:1
Mousaka batinjan	6.5	3.4	2.2	0.8	8.4	17.1	5	6.8	0.2	0.6	0.4	0.2:0.6:1
Riz a dajaj	5.4	2.2	2	0.9	6.9	11	4.7	7.2	0.4	0.9	0.4	7.2:0.4:1
Riz bi lahma	6.5	3.4	2.3	0.6	8.3	17.3	5.2	5.3	0.2	0.6	0.3	0.2:0.6:1
Sayadia	6.4	2.4	2.4	1.5	8.3	12.2	5.6	11.7	0.6	1	0.6	0.6:1:1
Shawarma dajaj	6.9	3.2	2.6	0.9	8.9	16.4	6	7.4	0.2	0.8	0.3	0.2:0.8:1
Shawarma lahma	8.2	4.9	2.7	0.4	10.6	24.8	6.2	3.2	0	0.5	0.1	0:0.5:1
Tabboula	4.2	1.7	2.3	0.1	5.4	8.7	5.2	1.4	0.1	1.3	0	0.1:1.3:1
Warak enab	3.9	2.4	1	0.4	5.1	12.3	2.3	3.1	0.1	0.4	0.3	0.1:0.4:1
Yakhnat Bamia	5.4	3.7	1	0.5	6.9	18.4	2.4	4.3	0.1	0.2	0.5	0.1:0.2:1
Yakhnat Fassoulia	3.9	2.6	0.8	0.3	5.0	13.3	1.9	2.5	0.1	0.3	0.3	0.1:0.3:1
Yakhnat Mouloukhia	4.2	2.5	1.3	0.4	5.4	12.5	2.9	3.2	0.1	0.5	0.3	0.1:0.5:1

SFA: saturated fatty acids; MUFA: monounsaturated fatty acids; PUFA: polyunsaturated fatty acids; P:S: polyunsaturated fatty acids: saturated fatty acids ratio; M:S: monounsaturated fatty acids: saturated fatty acids ratio; P:M: polyunsaturated fatty acids: monounsaturated fatty acids ratio; P:M:S: polyunsaturated fatty acids: monounsaturated fatty acids: saturated fatty acids ratio.

**Table 2:** Fatty acid ratios and total fatty acid mean values in 100 g of edible portions of Arabic sweets and the percentage of their daily contribution in a 2000 kcal-diet.

Arabic Sweet	In 100 g of Edible Portions (Per Gram)				Percentage of Daily Contributions in 2000 Kcal-Diet				Fatty Acid Ratios			
	Fat	SFA	MUFA	PUFA	Fat	SFA	MUFA	PUFA	P:S	M:S	P:M	P:M:S
Baklava mixed	23.4	12	9.4	1.9	30	92.8	21.4	8.7	0.1	0.7	0.2	0.1:0.7:1
Baklava mixed Light	20.5	10.7	8.8	0.8	26.2	53.6	20.1	3.8	0	0.8	0	0:0.8:1
Barazik	25.3	12.1	10.3	2.8	32.4	60.5	23.4	13.1	0.2	0.8	0.2	0.2:0.8:1
Boundoukia	16.5	14.2	1.9	0.2	21.1	71.1	4.4	1.1	0	0.1	0.1	0:0.1:1
Daoukia	19.5	4.2	13.6	1.5	25	21.1	31	7	0.3	3.2	0.1	0.3:3.2:1
Foustoukia	14.8	10.4	4.	0.2	18.9	52.1	9.2	1.1	0	0.3	0	0:0.3:1
Ghourayba	20.4	14	4	2.2	26.1	70.1	9	10.3	0.1	0.2	0.5	0.1:0.2:1
Halawa	25.8	16.1	7.9	1.5	33	80.6	18	7.1	0.1	0.4	0.2	0.1:0.4:1
Halawa light	25.5	12.6	12.2	0.5	32.6	63.1	27.8	2.3	0	0.9	0	0:0.9:0
Halawat El Jiben	8.9	7.2	1.3	0.2	11.4	55.8	3.1	0.9	0	0.1	0.1	0:0.1:1
Ish el bulbul	8.5	8.1	0.3	0	10.9	40.5	0.8	0.1	0	0	0.1	0:0:1
kallaj kashta	25.1	23.6	1.2	0.1	32.1	118.4	2.8	0.5	0	0	0.1	0:0:1
karabij joz maa crema	9.6	8.4	1	0.2	12.3	42	2.2	0.9	0	0.1	0.2	0:0.1:1
Katayef Kashta	6.6	4.3	1.9	0.3	8.5	33	4.4	1.5	0	0.4	0.1	0:0.4:1
knefe kashta with kaak	5.3	4.8	0.4	0	6.7	23.9	1	0.1	0	0	0	0:0:1
Kounafa bil jiben	12.2	11.1	0.9	0.1	15.7	85.3	2.2	0.4	0	0	0.1	0:0:1
Maakroun	12	9.4	2.2	0.3	15.3	72.3	5	1.5	0	0.2	0.1	0:0.2:1
Maakroun wa mshabbak	10.5	9	1.2	0.2	13.4	45.3	2.7	1	0	0.1	0.1	0:0.1:1
Maamoul tamer	17.4	11.5	5.2	0.5	22.3	88.4	11.8	2.6	0	0.4	0.1	0:0.4:1
Maamoul mad kashta	10.6	7.9	2.3	0.3	13.6	60.9	5.2	1.6	0	0.2	0.1	0:0.2:1
Maamoul mad joz	19.2	15.8	2.9	0.3	24.6	121.7	6.7	1.3	0	0.1	0.1	0:0.1:1
Maamoul fostok	14.4	12.2	2.1	0	18.4	60.9	4.8	0.3	0	0.1	0	0:0.1:1
Maamoul joz	21.5	12.1	6.8	2.3	27.5	93.1	15.6	10.5	0.1	0.5	0.3	0.1:0.5:1
Madlouka	19.2	8.6	7	3.4	24.6	43.2	16.1	15.8	0.4	0.8	0.4	0.4:0.8:1
Mafrouka kashta	13.2	9.9	2.5	0.7	16.9	76.3	5.7	3.3	0	0.2	0.2	0:0.2:1
Mafrouka fostok	10.6	7.4	2.6	0.4	13.5	37.1	6.1	1.9	0	0.3	0.1	0:0.3:1
Moushabak	20.1	11.9	7.1	0.9	25.7	59.7	16.3	4.1	0	0.6	0.1	0:0.6:1
Moufattaka	13.1	12.6	0.3	0	16.7	63.2	0.7	0.3	0	0	0.2	0:0:1
Mouhallabiya	20.7	2.5	8.1	10	26.5	12.5	18.4	45.7	4	3.2	1.2	4:3.2:1
Nammoura	5.9	4	1.4	0.3	7.5	30.8	3.3	1.4	0	0.3	0.2	0:0.3:1
Osmaliya	16.2	14.3	1.4	0.3	20.8	110.6	3.2	1.5	0	0.1	0.2	0:0.1:1
Riz bi halib	9.5	7.3	1.5	0.5	12.1	36.9	3.5	2.3	0	0.2	0.3	0:0.2:1
Saniora	23.8	16.6	6.1	0.6	30.5	128.1	14	3.1	0	0.3	0.1	0:0.3:1
Sfouf	12.4	8.2	3.5	0.5	15.9	63.3	8	2.2	0	0.4	0.1	0:0.4:1
Shaaybiyat	11.6	7.4	3.5	0.4	14.8	37.3	8	2.1	0	0.4	0.1	0:0.4:1
Ward el sham	16.1	8.7	6	1.3	20.6	43.5	13.7	6	0.1	0.7	0.2	0.1:0.7:1
Znoud El sitt	14.2	13	1	0	18.2	65.3	2.3	0	0	0	0	0:0:1

SFA: saturated fatty acids; MUFA: monounsaturated fatty acids; PUFA: polyunsaturated fatty acids; P:S: polyunsaturated fatty acids: saturated fatty acids ratio; M:S: monounsaturated fatty acids: saturated fatty acids ratio; P:M: polyunsaturated fatty acids: monounsaturated fatty acids ratio; P:M:S: polyunsaturated fatty acids: monounsaturated fatty acids: saturated fatty acids ratio.

**Table 3:** Fatty acid ratios and total fatty acid content in 100 g of edible portions of market food products and the percentage of their daily contribution in a 2000 Kcal-diet.

Food Groups	Product	In 100 g of Edible Portions (Per Gram)				Daily Contributions in 2000 Kcal-Diet				Fatty Acid Ratios			
		Fat	SFA	MUFA	PUFA	Fat	SFA	MUFA	PUFA	P:S	M:S	P:M	P:M:S
Cereals	Arabic Bread-White	2.3	0.6	0.4	1.2	2.9	3.2	0.9	5.5	1.8	0.6	2.8	1.8:0.6:1
	Arabic Bread-Whole wheat	4	1.3	0.5	2.1	5.1	6.9	1.1	9.6	1.5	0.3	4.2	1.5:0.3:1
	Baguette	0.5	0.1	Tr	0.2	0.6	0.7	0.1	1.2	1.7	0.4	4.3	1.7:0.4:1
	Breakfast Cereals	2.1	0.9	0.8	0.3	2.6	4.8	1.8	1.4	0.3	0.8	0.3	0.3:0.8:1
	Breakfast Cereals-Chocolate	2.4	2.1	0.2	Tr	3	10.5	0.4	0.3	0	0.1	0.4	0:0.1:1
	Croissant Zaatar-1	16.1	15.1	0.8	0.1	20.6	75.9	1.8	0.4	0	0	0.1	0:0:1
	Croissant zaatar-2	22.5	15.1	5.4	1.7	28.8	75.7	12.4	7.7	0.1	0.3	0.3	0.1:0.3:1
	Kaak Asrouni	1.5	0.2	0.5	0.7	1.9	1.1	1.1	3.4	3.2	2.1	1.5	3.2:2.1:1
	Kaak Debes and Cacao	11.9	5.5	4.9	1.3	15.2	27.9	11.2	6	0.2	0.8	0.2	0.2:0.8:1
	Kaak Korshalli	6.9	3.1	2.4	1.3	8.8	15.6	5.5	5.9	0.4	0.7	0.5	0.4:0.7:1
Oil and fats	Butter	81.4	56.4	23	1.4	104.3	282	52.3	6.6	0	0.4	0	0:0.4:1
	Butter Light	61.5	43.4	16.4	1.1	78.8	217.4	37.3	5	0	0.3	0	0:0.3:1
	Corn Oil	100	10.3	31	58.7	128.2	51.5	70.4	266.8	5.7	3	1.8	5.7:3:1
	Olive Oil	100	14.9	74.3	10.8	128.2	74.5	168.8	49	0.7	4.9	0.1	0.7:4.9:1
	Sunflower Oil	100	7.6	29.8	62.6	128.2	38	67.7	284.5	8.2	3.9	2.1	8.2:3.9:1
	Tahina	59.4	17.1	30	12.2	76.1	85.5	68.1	55.6	0.7	1.7	0.4	0.7:1.7:1
	Vegetable Margarine	99	46.7	27.4	24.8	126.9	233.6	62.3	112.9	0.5	0.5	0.9	0.5:0.5:1
Sugar	Biscuits Chocolate Quinoa	13.4	1.8	9.6	1.9	17.1	8.9	21.9	8.7	1	5.3	0.2	1:5.3:1
	Biscuits Digestive	17.1	15.6	1	0.3	21.9	78.2	2.3	1.7	0	0	0.3	0:0:1
	Biscuits Digestive Light	13.8	12.2	0.7	0.7	17.6	61	1.7	3.5	0	0	1	0:0:1
	Biscuits with cream	15.5	7.8	6	1.6	19.8	39	13.6	7.6	0.2	0.7	0.2	0.2:0.7:1
	Chocolate Dark	33.6	33.1	0.4	Tr	43	165.6	0.9	0.1	0	0	0	0:0:1.0
	Chocolate Milk-1	36.6	36.1	0.4	Tr	46.9	180.6	1	Tr	Tr	0	Tr	0:0:1.0
	Chocolate Milk-2	35	34.7	0.2	Tr	44.8	173.6	0.5	0.1	0	0	0.1	0:0:1.0
	Doughnuts	19.6	12.8	6	0.6	25.1	64.3	13.6	2.7	0	0.4	0.1	0:0.4:1
	English Cake-Chocolate	18.6	9.17	6.4	2.4	23.8	45.8	14.7	11.1	0.2	0.7	0.3	0.2:0.7:1
	Cake with Cream	16.1	14.2	1.4	0.3	20.6	71.3	3.3	1.6	0.	0.1	0.2	0:0.1:1
	Pain au Lait	3.8	1.9	0.9	0.8	4.8	9.8	2	3.7	0.4	0.4	0.9	0.4:0.4:1
	Petit Fours-1	25.6	15.9	8.4	1.2	32.8	79.4	19	5.7	0	0.5	0.1	0:0.5:1
	Petit Fours-2	29.6	26.7	2.6	0.1	37.9	133.9	5.9	0.5	0	0.1	0	0:0.1:1
	Wafer-Chocolate-1	21.7	19.6	1.4	0.5	27.8	98.4	3.2	2.6	0	0	0.4	0:0:1
	Wafer-Chocolate-2	24.2	19.1	2.4	1	31	95.5	5.5	4.8	0	0.1	0.4	0:0.1:1
Drinks	Coffee with cardamom	16.8	14	2.1	0.5	21.5	70.3	4.7	2.6	0	0.1	0.2	0:0.1:1
	Coffee without cardamom	17.7	12.9	2.5	2.1	22.6	64.6	5.8	9.8	0.1	0.2	0.8	0.1:0.2:1
	Hot Chocolate Powder	5.4	3.5	1.7	0.1	6.9	17.5	3.8	0.8	0	0.4	0.1	0:0.4:1
	Instant Coffee	10.8	10.6	0.1	Tr	13.8	53.3	0.2	0.1	0	0	0.2	0:0:1
Nuts and seeds	De-hulled pumpkin seeds	50.6	34	15.4	0.8	64.8	170.2	35	3.6	0	0.4	0	0:0.4:1
	De-hulled sunflower seeds	52.5	26.7	19.1	6.3	67.3	133.6	43.4	28.6	0.2	0.7	0.3	0.2:0.7:1
	Mixed Nuts	25.7	13.1	10.1	2.3	32.9	65.9	23	10.5	0.1	0.7	0.2	0.1:0.7:1
	Mixed Kernels	53.6	10.9	39.4	3.1	68.7	54.9	89.6	14.3	0.2	3.5	0	0.2:3.5:1
Snacks	Potato Chips-1	29.9	20.2	9.5	0.1	38.3	101.2	21.6	0.5	0	0.4	0	0:0.4:1
	Potato Chips-2	15.4	6.5	7.9	0.9	19.7	32.4	17.9	4.3	0.1	1.2	0.1	0.1:1.2:1
	Potato Chips Light-1	26.9	18.3	8.4	Tr	34.4	91.8	19.2	0.2	0	0.4	0	0:0.4:1
	Potato Chips Light-2	22.9	15	6.9	0.8	29.3	75.2	15.8	3.7	0	0.4	0.1	0:0.4:1
Canned	Tuna Packed in Oil	6.8	3.2	2	1.4	8.7	16.3	4.6	6.6	0.4	0.6	0.7	0.4:0.6:1
	Tuna Packed in Water	0.5	0.2	0.2	Tr	0.6	1.2	0.5	0.1	0.1	0.9	0.1	0.1:0.9:1

SFA: saturated fatty acids; MUFA: monounsaturated fatty acids; PUFA: polyunsaturated fatty acids; P:S: polyunsaturated fatty acids: saturated fatty acids ratio; M:S: monounsaturated fatty acids: saturated fatty acids ratio; P:M: polyunsaturated fatty acids: monounsaturated fatty acids ratio; P:M:S: polyunsaturated fatty acids: monounsaturated fatty acids: saturated fatty acids ratio.

**Table 4:** List of fat composition of food commonly tested in Lebanon, Kuwait, Jordan, Saudi Arabia, and Bahrain [22,23,24,25,26,27].

Comparison with EM Countries	[24]	[25]	Lebanon 2020	[27]	[22]	[23]	[26]
Dishes Names	Fat in 100 g	Fat in 100 g	Fat in 100 g	Fat in 100 g	Fat in 100 g	Fat in 100 g	Fat in 100 g
Baba ghanouj	3.7	NA *	9.44	8.7	5.4	NA	NA
Batata mahshi	5.6	NA	1.24	NA	NA	5.9	NA
Bourghol bil banadoura	NA	10.16	5.02	NA	NA	NA	NA
Chichbarak	NA	8.89	4.6	NA	NA	NA	NA
Falafel	NA	NA	11.7	NA	18.4	14.3	NA
Fatayir sabanekh	6.6	NA	11	6.12	7.6	2.75	7
Fattoush	6.3	NA	2.94	2.17	8.6	NA	NA
Foul moudamas	3.1	NA	3.48	3.15	7.3	3.2	NA
Hindbe bil zeit	6.7	NA	10.7	NA	NA	NA	NA
Hommus bil tahina	19.7	NA	6.4	7.7	NA	17.8	NA
Mosakaa batinjen	NA	NA	6.58	NA	NA	16.4	NA
Kafta (only)	22.1	NA	6.32	NA	NA	NA	NA
Kussa mahshi	1.7	NA	2.42	NA	NA	NA	NA
Lahm bil ajin	39.5	NA	8	NA	NA	NA	NA
Malfuf mahshi	2.6	NA	2.12	NA	NA	NA	NA
Moujadara	5.6	NA	5.8	NA	NA	NA	NA
Riz a dajaj	9.3	NA	5.4	NA	NA	NA	NA
Sayadiya	13.2	NA	6.48	3.98 (sandwich)	NA	NA	NA
Shawarma dajaj	NA	NA	6.94	3.90 (sandwich)	NA	NA	11.2
Shawarma Lahma	36	NA	8.28	NA	14	NA	9.4
Tabbouli	5.8	NA	4.24	3.3	2.6	NA	NA
Warak enab mahshi	7.3	NA	3.98	NA	NA	3.7	NA
Yakhnat bamyah	7.2	11.09	5.4	NA	NA	NA	NA
Yakhnat Fassoulia	6.6	NA	3.9	NA	NA	NA	NA
Yakhnat mulukhiya	6.4	NA	4.28	NA	NA	0.25	NA
Baklava mixed	NA	NA	27.3	NA	28.7	NA	38.8
Barazik	NA	NA	42.1	NA	31.6	NA	NA
Ghouraybah	21.3	NA	32.9	NA	28	NA	NA
Kallaj kashta	NA	NA	9.7	NA	25.8	NA	NA
Katayef kashta	NA	NA	10.5	NA	7.1	NA	NA
Knefah bil jibn	NA	NA	13.2	NA	21.6	NA	NA
Maakroun and Moushabak	17.2	NA	17.9	NA	NA	NA	NA
Maamoul tamer	9	NA	15.6	NA	16.3	NA	NA
Maamoul fostok	NA	NA	26.9	NA	19.3	NA	NA
Maamoul joz	NA	NA	18.3	NA	27.3	NA	NA
Nammoura	3.1	NA	8.8	NA	18.4	NA	NA

* NA: Not available.

**Table 5:** Comparison between actual nutrient analysis values of market food products and reported nutrient values on nutrition fact label.

Product	Fat		SFA		MUFA		PUFA	
	Chem-R *	N.label ^	Chem-R	N.label	Chem-R	N.label	Chem-R	N.label
Biscuits Chocolate Quinoa	13.4	5	1.8	Tr	9.6	NA	1.9	NA
Biscuits Digestive	17.1	26	15.6	15	1	NA	0.3	NA
Biscuits Digestive Light	13.8	13.5	12.2	13.5	0.7	NA	0.7	NA
Breakfast Cereals	2.1	0.4	0.9	0.1	0.8	0.1	0.3	0.2
Breakfast Cereals-Chocolate	2.4	5.2	2.1	5.2	0.2	NA	0	NA
Butter	81.4	80	56.4	54	23	NA	1.4	NA
Butter Light	61.5	20	43.4	53.3	16.4	NA	1.1	NA
Chocolate Wafers	21.7	25	19.6	21	1.4	NA	0.5	NA
Chocolate wafers-brand 2	24.2	27.27	19.1	9	2.4	NA	1	NA
Chocolate with milk	36.6	73	36.1	46	0.4	NA	Tr	NA
Chocolate-Dark	33.6	40	33.1	23.3	0.4	NA	Tr	NA
Chocolate-White	35	NA	34.7	NA	0.2	NA	Tr	NA
Corn Oil	100	100	10.3	14	31	24	58.7	61
Cream Filled Biscuits	15.5	18.6	7.8	4.6	6	NA	1.6	NA
Hot Chocolate Powder	5.4	5.9	3.5	3.7	1.7	NA	0.1	NA
Instant Coffee (3 in 1)	10.8	9.09	10.6	9	0.1	NA	0	NA
Chips Baked	22.9	22	15	6	6.9	NA	0.8	NA
Lays Chips Cheese	15.4	12	6.5	3	7.9	NA	0.9	NA
Olive Oil	100	100	14.9	24	74.3	87	10.8	22
Potato Chips	29.9	26.6	20.2	10	9.5	10	0.1	3.3
Potato Chips Light	26.9	20	18.3	6.6	8.4	6.6	Tr	NA
Sunflower Oil	100	100	7.6	10	29.8	20	62.6	60
Tahina	59.4	60	17.1	10	30	NA	12.2	NA
Tuna Packed in Oil	6.8	26.6	3.2	NA	2	NA	1.4	NA
Tuna Packed in Water	0.5	3	0.2	NA	0.2	NA	Tr	NA
Vegetable Margarine	>99	56.4	46.7	9.5	27.4	19	24.8	23

* Chemical results, ^ nutrition facts label, NA: not available, Tr: trace, SFA: saturated fatty acids; MUFA: monounsaturated fatty acids; PUFA: polyunsaturated fatty acids.

## Data Availability

All the study data are reported in this paper.
